# Advance care planning in glioblastoma patients: development of a disease-specific ACP program

**DOI:** 10.1007/s00520-019-04916-9

**Published:** 2019-06-26

**Authors:** Lara Fritz, Hanneke Zwinkels, Johan A. F. Koekkoek, Jaap C. Reijneveld, Maaike J. Vos, Linda Dirven, H. Roeline W. Pasman, Martin J. B. Taphoorn

**Affiliations:** 1 Department of Neurology, Haaglanden Medical Center, PO BOX 2191, 2501 VC The Hague, The Netherlands; 2 Department of Neurology, Leiden University Medical Center, Leiden, The Netherlands; 3 Department of Neurology and Brain Tumor Center Amsterdam, Amsterdam University Medical Centers (location VUmc), Amsterdam, The Netherlands; 4 Department of Neurology, Amsterdam University Medical Centers (location Academic Medical Center), Amsterdam, The Netherlands; 5 Department of Public and Occupational Health, Amsterdam Public Health Research Institute, Expertise Center for Palliative care Amsterdam, Amsterdam University Medical Centers, Vrije Universiteit Amsterdam, Amsterdam, The Netherlands

**Keywords:** Advance care planning, Glioblastoma, End of life care, Brain tumor, Health-related quality of life

## Abstract

**Background:**

It is unknown if the implementation of an advance care planning (ACP) program is feasible in daily clinical practice for glioblastoma patients. We aimed to develop an ACP program and assess the preferred content, the best time to introduce such a program in the disease trajectory, and possible barriers and facilitators for participation and implementation.

**Methods:**

A focus group with health care professionals (HCPs) and individual semi-structured interviews with patients and proxies (of both living and deceased patients) were conducted.

**Results:**

All predefined topics were considered relevant by participants, including the current situation, worries/fears, (supportive) treatment options, and preferred place of care/death. Although HCPs and proxies of deceased patients indicated that the program should be implemented relatively early in the disease trajectory, patient-proxy dyads were more ambiguous. Several patient-proxy dyads indicated that the program should be initiated later in the disease trajectory. If introduced early, topics about the end of life should be postponed. A frequently mentioned barrier for participation was that the program would be too confronting, while a facilitator was adequate access to information.

**Conclusion:**

This study resulted in an ACP program specifically for glioblastoma patients. Although participants agreed on the program content, the optimal timing of introducing such a program was a matter of debate. Our solution is to offer the program shortly after diagnosis but let patients and proxies decide which topics they want to discuss and when. The impact of the program on several patient- and care-related outcomes will be evaluated in the next step.

**Electronic supplementary material:**

The online version of this article (10.1007/s00520-019-04916-9) contains supplementary material, which is available to authorized users.

## Introduction

With an annual incidence of approximately 3 per 100,000 persons, glioblastoma is the most common type of glioma and also the most severe subtype [1, 2]. Patients have a median survival of only 15 months, despite multimodal treatment with surgery, radiotherapy, and chemotherapy [3].

During the course of the disease, glioma patients may experience progressive neurological deficits, such as motor deficits, seizures, and cognitive dysfunction [4–7]. Progressive cognitive decline may seriously interfere with patients’ ability to make decisions regarding treatment or care [8–10]. It therefore seems important to involve glioma patients early in the disease trajectory in treatment decision-making [11]. A way to achieve this is with advance care planning (ACP). ACP is a process to involve patients and their proxies at an early stage in decision-making on future (palliative) care, including end of life (EOL) care [12]. ACP allows patients and their proxies (defined as persons that are involved in the patient’s care trajectory, e.g., partner, spouse, child, parent, neighbor), together with their physicians, to evaluate all care options and to communicate their preferences. However, it is unclear what the optimal timing of introduction of such a program is [13–15].

An increasing body of evidence suggests that early palliative care is effective in improving mood and health-related quality of life (HRQoL) of cancer patients in their EOL phase [16, 17]. ACP could be part of such an early palliative care trajectory. Furthermore, ACP has shown to improve outcomes in older patients and patients with chronic diseases, including patient/family satisfaction and the quality of EOL care, as well as a reduction in stress, anxiety, and depression in surviving proxies [18]. Also, the level of agreement between the patients’ preference for care and the actually received care increased [19].

It has been suggested that early palliative care through structured ACP, focusing on topics such as timely identification and treatment of disease-specific symptoms, could improve HRQoL of glioblastoma patients as well as symptom control [20]. Other studies showed that the majority of glioblastoma patients died at their preferred place if this was expressed [21], which was associated with increased dignity [22]. Moreover, if high-grade glioma patients expressed their EOL care preferences, these were often met (90%) [23]. Thus, ACP could potentially improve HRQoL and quality of care in glioblastoma patients [24], and a program addressing the specific needs of glioblastoma patients is warranted.

Although promising, it is unknown if the implementation of an ACP program would be feasible in daily clinical practice for glioblastoma patients. Before implementing such a program, this should be developed in such a way that it meets the needs of glioblastoma patients and their proxies [11]. The aim of this study was to develop an ACP program specifically for glioblastoma patients. To do so, we evaluated topics that are relevant for patients and their proxies, as well as practical issues including the appropriate timing of initiation of such a program in the disease trajectory, and facilitators and barriers to participate in an ACP program.

## Methods

### Participants

Eligible patients were adults with a histologically confirmed glioblastoma (WHO grade IV) or molecularly glioblastoma-like tumors in any stage of their disease, and/or their proxies, who visited the outpatient clinic of a large tertiary hospital (Haaglanden Medical Center, The Hague, The Netherlands, accredited as a national center of expertise for glioma patients) between September 2016 and January 2017. Patients were excluded if they did not understand the Dutch language, judged as incompetent by their treating physician (i.e., lacking capacity to consent to research), or if they previously received formal ACP. All possibly eligible patients were identified by the researcher prior to their consultation and they were asked for participation by their treating physician. In case they did not want to participate, several patient- and disease-related characteristics were recorded to assess selection bias. Moreover, healthcare professionals (HCPs) specialized in brain tumors and/or palliative care were invited to participate, as well as proxies of deceased patients (they were invited approximately 3 months after the patient’s death).

### Data collection

A focus group with HCPs was conducted, as well as semi-structured interviews with patient-proxy dyads (i.e., a group of two people, in this case, the patient together with his/her proxy), a patient or proxy alone, and proxies of deceased patients. During the focus group/interviews, three topics were discussed: the preferred content of the ACP program, the optimal timing to introduce such a program, and barriers and facilitators to participate in an ACP program (an overview of the topics discussed during the semi-structured interview are presented in Supplementary File 1). The focus group was moderated by one researcher (LF), who did not participate in the discussions during the session. The semi-structured interviews were conducted by the same researcher (LF). No other HCPs were involved in the interviews, nor as being an interviewer, nor as being interviewed. Both the focus group and individual conversations were recorded.

### Content semi-structured interviews

#### Content of ACP program

The topics discussed in the focus group and semi-structured interviews were based on a literature search and expert opinion (i.e., research team). Topics were categorized into (1) current situation, (2) worries and fears of the patient and proxy, (3) (supportive) treatment options, and (4) preferred place of care and death (Table 1). All participants were asked to indicate whether they regarded the presented topics as important to include in the ACP program, and if topics were missing.

**Table 1:** Topic list for ACP program used in focus group and interviews

Category	
Current situation	Current health issues, future perspective, resources (psychological support, etc.), relationship with family/friends
Worries and fears	Anxiety and worries of patients and proxies, concerns with respect to performing household or work, etc.
(Supportive) treatment	Treatment preference and goals of care form, substitute decision maker, anti-tumor treatment, supportive treatment, supportive treatment in the EOL phase, withdrawal and withholding of treatment, palliative sedation, and euthanasia
Preferred place of care and death	(Im)possibilities for place of care and death

#### Timing to introduce ACP

During the focus group, HCPs were requested to indicate the best moment in the disease trajectory to offer an ACP program to patients: (1) shortly after diagnosis, (2) after chemoradiation, about 12–16 weeks after diagnosis, (3) after the first three courses of adjuvant chemotherapy, approximately 6 months after diagnosis, (4) after adjuvant chemotherapy, about 9 months after diagnosis, or (5) another moment. These options were also presented to patients/proxies (this may have been hypothetical for some, as they were not in that disease stage yet), and proxies of deceased patients.

#### Barriers and facilitators to participate in an ACP program

Possible barriers (negative influences) and facilitators (positive influences) for participation and implementation were experienced which could be considered when optimizing implementation of and participation in the ACP program.

### Data analysis

Descriptive statistics were used to describe the participant characteristics, using IBM SPSS version 21.0. The semi-structured interviews and focus group were transcribed verbatim and analyzed thematically using the software program NVIVO 11. To assure reliability of coding, all transcripts of the interviews and focus group were coded by two researchers, and differences were resolved in consensus.

## Results

### Participants

#### Focus group

The focus group consisted of neurologists specialized in neuro-oncology (*n* = 2), a nurse specialist in neuro-oncology (*n* = 1), a nurse specialist in oncology (*n* = 1), radiation oncologists specialized in neuro-oncology (*n* = 2), a palliative nurse of a hospice (*n* = 1), a general practitioner (*n* = 1), a nursing home physician (*n* = 1), and a senior researcher with expertise in EOL care in different patient populations (*n* = 1).

#### Patients and proxies

We aimed to include at least five patient-proxy dyads and five proxies of deceased patients. In total, 13 patient-proxy dyads and six proxies of deceased patients were invited for participation.

Eight out of the 13 patient-proxy dyads participated. Of note, one interview was held with the patient only and one with the proxy only, and one patient-proxy dyad was interviewed separately. Most participating patients were male, with a mean age of 61, and all had a good performance status (KPS ≥ 70). Although most patients were diagnosed with glioblastoma, one patient with a molecularly glioblastoma-like tumor was included. Most proxies were female and their mean age was 57 years.

Six proxies of deceased patients were invited, of which five participated in the semi-structured interviews. Their mean age was 68 years. Proxies of deceased patients were invited to participate after a median of 6 months (range 3–7) after the death of the patient. Most of the deceased patients were male, with a mean age of 68 at the time of death, see Table 2 for the baseline characteristics of the participants.

**Table 2:** Characteristics of patients, their proxies, and proxies of deceased patients

	Living patients *N = 8*	Proxies of living patients *N = 8*	Proxies of deceased patients *N = 5*
Age in years at time of interview, mean (SD)	61 (8)	57 (10)	68 (7)
Gender, no.			
Male	6	1	1
Female	2	6	4
Not participating		1	
Karnofsky performance status (KPS)			
Median (range)	80 (80–90)	N/A	N/A
KPS ≥ 70, no.	8		
Month since diagnosis			
Median (range)	12 (3–69)	N/A	N/A
Current anti-tumor treatment, no.			
No	4	N/A	N/A
Yes	4		
Recurrent disease, no.	3	N/A	N/A
Current and previous treatment		N/A	N/A
Previous initial			
Resection	8		
Chemotherapy	6		
Radiotherapy	7		
Other	0		
Previous recurrence			
Resection	1		
Chemotherapy	2		
Radiotherapy	1		
Other	2		
Current			
Chemotherapy	2		
Radiotherapy	1		
Other	2		
Highest level of education†, no.			
Lower education	1	2	3
Medium education	1	3	1
High education	6	2	1
Missing	0	1	0
Relation to the patient, no.			
Partner	N/A	6	5
Child		1	0
Not participating		1	0
Duration of the relationship (in years), mean (SD)		40 (9)	40 (16)
Contact intensity, no			
Living together	N/A	7	5
Daily		1	0
Weekly		0	0
Monthly		0	0
Religious, no			
No	3	1	3
Yes	5	6	2
Missing	0	1	0
If yes, religion important, no.			
	2	4	2

*Months between diagnosis and death

^†^Level of education is based on The International Standard Classification of Education (ISCED). Scores range between 1 and 8, with a higher score representing a higher level of education. Scores 1–2 are classified as a low level of education, scores 3–5 as a medium level of education, and scores 6–8 as a high level of education

*N/A*, not applicable

Patients who participated in the study were not different from the patients who were eligible but declined to participate with respect to gender, tumor type, age, and performance status (data not shown).

#### Reasons not to participate

Five out of thirteen patient-proxy dyads who were invited for participation declined. There were various reasons for non-participation including a poor physical condition, being too early to talk about such topics, not wanting to think about these topics, considering this type of conversation too confronting, already having too much on their mind, or considering the program non-beneficial because everything was already arranged.

### Content of ACP program

All participants, i.e., focus group participants, patient-proxy dyads, and proxies of deceased patients, were requested to indicate whether the proposed topics were relevant to include in the ACP program. The focus group deemed the topics of all themes as relevant. All topics within the theme “current situation” were also considered important by the patient-proxy dyads and proxies of deceased patients, particularly future perspective and the relationship with family/friends. Within the theme “worries and fears,” all topics were deemed important to include. In addition, new topics that emerged were the needs of proxies and financial problems. In general, all topics within the theme “(supportive) treatment” were found to be important by participants. One proxy of a deceased patient said she rather did not want to talk about euthanasia. Lastly, all topics within the theme “preferred place of care and death” were deemed important to discuss. However, one patient and one proxy indicated that these topics should only be discussed when the moment was there, see Table 3 for quotes of participants emphasizing the relevance of the proposed topics.

**Table 3:** Quotes of patients, proxies, or proxies of deceased patients regarding the relevance of the topics to be included in the ACP program

Topic	Relevant quote of participant
Current sitiation	
Current health issues	*Interviewer: “Do you think it is important to ask about the current situation? How are you? Do you have complaints?”* *Proxy: “Yes, I just wanted to say, things like how are you? What are the problems? What do you think has changed?”*
Future perspective	*Proxy: “Yes, you have to be honest and you need to know when the doctors think that you are entering the last phase of life. Because you must arrange practical things.”*
Resources (psychological support, etc.),	*Proxy: “It may be that, yes, that one of us, well we are rather open to each other. I mean, I ask my questions to [patient] and do not think, oh I cannot really ask this. And* vice versa *too. But if the situation is not like that, that is also possible, you can as a patient or as a partner, you can remain with a lot of questions you cannot ask, and that may cause a lot of stress, I think.”Patient: “Yes”Proxy: “At that time I think it is very good that you know, I can still go to someone else, an objective person.”*
Relationship with family/friends	*Patient: “Yes, I think that what you are constantly confronted with, is the environment, they often have, they have of course also have delved into your situation, so everyone finds information everywhere and says to you can also do this or whatever. Or there is another article in the newspaper or whatever. Another new therapy for, what is it cancer or whatever. And then I get that article printed out on my desk so to speak, so to speak. You know, that kind of thing.”*
Worries and fears	
Anxiety	*Proxy: “Yes, yes, fear of what is coming, yes. I think, yes, you have to open it for discussion, certainly for the patient, I feel that way.”*
Household, work, etc.	*Patient: “Yes, I think, how do you say that? Ultimately it is just the future. What is the future going to bring and what is [..], your income and things like that. Because in the end you will be in a situation where you are no longer fully employable, at work or wherever. And that means a bit of a decrease in your income.”*
Worries of proxies	*Proxy: “But yes, in that situation you have to continuously weigh things, and you also have to consider what the children can handle. [..] because at some point, you get a huge field of tension, while you have to keep functioning at work and your family.”*
Worries of patients	*Patient: “Yes, that is very important. I am now, I am now with a psychologist. And that is just wonderful, even if people think, what do you do have to do there. But it’s just nice to be with such a person because when you are over, you can just let it go, everything.”*
(Supportive) treatment	
Preference and goals form	*Patient: “But very well. The more, the more clarity you have, the more you will be able to determine things yourself, officially documented.”*
Substitute decision maker	*Proxy: “Yes I think that that is very personal.”Patient: “Yes”Proxy: “Because that person might do things that the partner does not, as a matter of speech.”* *Patient: “No, in that case it is good that you obviously have mentioned that beforehand, or that it is included in the program.”*
Palliative sedation	*Proxy: “I mean, say about 30 years ago this did not really exist, it was not done. But nowadays there is simply more possible, yes, and then you should have the patient decide what he wants to do with it.”*
Euthanasia	*Proxy of deceased patient: “Yes, it is, I cannot remember if someone had said anything to us about euthanasia. I do not know if I would have liked that. On the other hand, it is not about me. So that, it is difficult.”* *Proxy: “At the moment there is no longer good quality of life, and she says, well I just really do not want this anymore. I think euthanasia is very important.”*
Withdrawal and withholding of treatment	*Interviewer: “Suppose that someone with epilepsy can no longer swallow, that we can reassure people in advance that they do not have to worry about this, but that we can tell them what to do when something like this occurs.”Proxy of deceased patient: “Yes, I asked [nurse] this over the phone at that moment. What did not work anymore. That’s what she said, there are still a few drops, but that lasted very briefly, because I believe a day or 3 later the doctor has canceled everything.”*
Supportive treatment in the end of life phase	*Proxy: “In fact you are almost facing the choice, are you going to extend your life or do not you not want to extend life?Interviewer: “Yes what does someone want?”* *Proxy: “Yes, yes.” I think that something is just, I think, just an issue when it comes to that. And I think that there are not a lot of people who think that that can happen. Because there are of course all kinds of things that can be done.”*
Anti-tumor treatment	*Patient: “You just know, okay, with this type of medication, I might be able to continue for a bit longer, but side effects of these drugs may be that I can only lie in bed, so to speak. So yes, that is not what I want, just saying. That you can make a clear choice between them.” *Proxy of deceased patient: [..] And that is a very difficult conversation. How shall I say that? I mean, if you assume that you know how it will look like in the end, and you know she is dying, then the question is, how do you want to die? And of course, there are a lot of people who do not think about that.” I think one of the problems that exists is, of course, is that a lot of people have trouble thinking about the idea that they should stop treatment. And at the same time, of course, it also has to do with the risks, but also the side effects of a treatment, and if patients still have their dignity.”
Preferred place of death	
Possibilities for place of care and death	*Proxy: “But you can only make a decision if you have knowledge of course.”Interviewer: “That is what we want to accomplish with the program.”Proxy: “Exactly.”Interviewer: “Discuss, discuss.”Proxy: “So many possibilities and from there..”. *Interviewer: “Do you think that is important to include this in the program?”Proxy: “I think that it is important, but I think that it is, yes, something that you are not going to discuss in this phase of the disease.”
Possibility that preferred place of care/death is not possible	*Proxy of a deceased patient: “I think this is a very important topic. Because, actually you are a layman, and you do not know what to expect. So I think it is important to discuss this. For the relatives, what they are facing and what they can do.”*

### Timing of ACP program

**Fig. 1:**
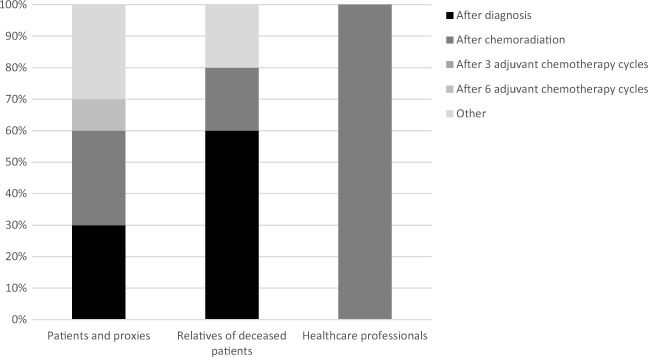
Preference for the moment in the disease trajectory when the ACP program should be implemented

The HCPs decided, in consensus, that after chemoradiation would be the most optimal moment to offer ACP to patients. The main reason was that patients are still competent early in the disease trajectory and therefore generally able to communicate their wishes. Proxies of deceased patients indicated that the program should be offered as early as possible during the disease course; shortly after diagnosis or after chemoradiation (Fig. 1). This would prepare them sufficiently for the future.

The patients and their proxies, however, were more ambiguous. Although most patient-proxy dyads indicated that the optimal timing was early in the disease trajectory (after diagnosis or after chemoradiation), other patient-proxy dyads indicated that ACP should be offered later in the disease trajectory (after six adjuvant chemotherapy cycles or at the time that anti-tumor treatment is no longer meaningful). Of note, all choices reflected moments the patients/proxies already had experienced in the disease trajectory, indicating that they felt that those moments were appropriate.

### Barriers and facilitators

#### Facilitators

Both patient-proxy dyads and proxies of deceased patients mentioned obtaining information, sufficient time to discuss issues, providing peace, discussing wishes, availability of an appropriate conversational partner, and the possibility to discuss certain topics as facilitators to participate in an ACP program. Other facilitators were that everything will be organized in advance, guidance during the disease trajectory, and face-to-face contact with a conversational partner and a fixed contact person.

Patient-proxy dyads also mentioned that participating in an ACP program may facilitate informing all involved disciplines on their wishes, that patients and their families are forced to think about these topics, that  patients can stay in control, that proxies are strongly involved, and that help can be found timely.

Facilitators specifically mentioned by HCPs were letting patients choose the topics, patients’ awareness of the situation, and a nurse who has enough time to discuss these topics. Similar to proxy dyads and proxies of deceased patients, HCPs mentioned that facilitators to participate in an ACP program could be providing hope and/or peace, providing information, the possibility to discuss certain topics, giving patients the opportunity to arrange everything in advance, to stay in control, and a fixed contact person during the disease trajectory.

#### Barriers

Both patient-proxy dyads and proxies of deceased patients mentioned that not wanting to think about the proposed topics would be a barrier to participate in an ACP program. Similarly, HCPs said that patients and their proxies might not want to discuss these topics with them. Other barriers mentioned by patients/proxies were difficulties in communicating with the patient, that it may be too confronting to talk about, or overwhelming, and more practically, that it might be difficult to participate in such a program with their proxy, or that it might not be helpful if they did not have a strong connection with their conversational partner.

Patient-proxy dyads further indicated that possible barriers were that participating might cost too much time and energy and that it requires traveling to the hospital, but also that they do not want to know everything. Lastly, HCPs indicated that this type of conversations might damage the physician-patient relationship.

## Discussion

In this study, we aimed to develop an ACP program and assessed the preferred content of the program, the best time to introduce such a program in the disease trajectory, and possible barriers and facilitators for participation and implementation.

Participants considered all predefined topics important to discuss in an ACP program. Besides, two new topics were proposed, being the needs of proxies and financial problems. Financial problems have been recognized as important in previous studies [21] and are often included as a topic in need assessment questionnaires. Moreover, (practical) needs of proxies have been identified as important [25–28]. Indeed, the caregiver burden is found to be high and associated with cognitive deterioration of patients and changes in their personality and behavior [29]. Previous research showed that in the EOL phase, approximately 50% of caregivers indicated a high burden and feelings of stress [30]. Reasons were changes in the relationship between the patient and caregiver, an adaptation of new roles and new responsibilities [31]. Providing information and concrete advice on dealing with these everyday difficulties can benefit caregivers [32].

It has been suggested that structured ACP may provide better symptom control (through early identification of symptoms and treatment) and improvement of psychosocial support and EOL planning in brain tumor patients [20]. Our results confirm the importance of these topics. Moreover, a previous study showed that most brain tumor patients are willing to discuss ACP issues [33]. Patients in our study who were not willing to discuss EOL issues indicated that these were discussed too early, and should only be discussed when becoming relevant (i.e., if they enter the EOL phase). Whereas HCPs and proxies of deceased patients indicated that the ACP program should be offered relatively early in the disease trajectory, with proxies of deceased patients indicating that the program should even be introduced as soon as possible (i.e., shortly after diagnosis), patient-proxy dyads were more ambiguous. Several patient-proxy dyads indicated that the program should be initiated later in the disease trajectory, after adjuvant chemotherapy or even when the moment is there (i.e., at the start of the patient’s EOL phase). This is in line with previous findings that brain tumor patients did not want to discuss ACP issues shortly after diagnosis [33]. It has been suggested that it might even be inappropriate to initiate ACP during active treatment [34] because it may take away the hope of patients and their relatives. Uncertainty about the disease trajectory and lack of information regarding prognosis may influence how patients perceive the introduction of ACP [33]. In another study, the majority of palliative care patients, including cancer patients in all disease stages, felt that the best timing of introducing ACP was after disease recurrence, failure of curative treatment, or at the time it became evident that they had a poor prognosis [34]. In contrast, a study including terminally ill patients found that the best timing of introducing early palliative care was soon after diagnosis [17]. Since the timing to discuss ACP has an impact on its acceptability and effect [14, 34], it is important to choose the most appropriate moment in the disease trajectory. Although several patients in our study indicated that early implementation of ACP is not preferred, it should be considered that glioblastoma patients have an incurable disease and may experience a rapid decline in their cognition, hampering decision-making later in the disease process [9, 10]. Timely initiation of ACP seems therefore warranted in this population. Considering the preferences of all participants, the most optimal moment to introduce the ACP program seems to be relatively early in the disease trajectory, i.e., for glioblastoma patients right after chemoradiation, although the best timing remains a matter of debate, also in other diseases (e.g., dementia) [14, 21, 35, 36]. However, to meet the wishes of patients/proxies, we propose that they should be able to choose the topics they want to discuss as well as the depth of the conversation. Nevertheless, by presenting issues that could become relevant in the future, we hope to trigger patients to at least think about these topics.

Barriers and facilitators affect the acceptance of an ACP program and should therefore be considered when designing and implementing an ACP program for glioblastoma patients. In this study, an important facilitator was that it offers patients a way to timely discuss and plan their future care, thereby empowering patients and proxies. The presence of a trained neuro-oncology nurse was also believed to facilitate this process and has been acknowledged previously [33]. In contrast, participating in such a program may also be confronting for patient/proxies, thereby being an important barrier. A barrier for general practitioners (GPs) to initiate ACP in cancer patients was the limited information provided by specialists in the hospital, as well as limited contact with their patients and the continuation of anti-tumor treatment by the treating physician even when this was no longer relevant [37]. By initiating ACP in the hospital and stimulating to draft a form with the patients’ wishes and goals of care, and subsequently communicating these to the GP, the cooperation between different healthcare professionals, as well as the quality and continuity of the patient’s care, may be improved. We therefore aim to include completion of a patients’ wish form in the ACP program.

Although the results of this study may guide further research on the implementation of ACP in clinical practice for glioblastoma patients, several limitations should be considered. First, participants were from the Netherlands, and results may be different for patients in other countries, especially due to cultural and religious differences [35, 38, 39]. One example is the inclusion of the topic euthanasia, which is only applicable to certain countries [40]. Second, particularly patients with an interest in ACP may have participated in this study, hampering generalizability of the results. This also holds true for clinical practice, in which it is likely that not all patients and/or proxies are willing to participate. Nevertheless, we suggest offering this program to all patient-proxy dyads visiting the outpatient clinic, so they will know that this type of care is available whenever required. Lastly, with respect to the applied methodology, the relevance of topics was not assessed using a validated scale (e.g., Likert scale assessing the strength of relevance), but rather as “relevant” or “not relevant.” However, patients were encouraged to explain their choice. Also, not all specialists involved in the care of glioblastoma patients participated in the focus group (e.g., medical oncologist and neurosurgeon), which may have hampered the identification of all relevant issues.

In conclusion, ACP appears important for glioblastoma patients, and currently, no evidence-based ACP program specifically for this patient population exists [11, 20, 24]. The results of this study are used to develop an ACP program that meets glioblastoma patients’ needs. In addition, a strategy to implement this program will be developed. A next step would be to offer this program to glioblastoma patients and their proxies and to evaluate the impact of this program on outcomes such as HRQoL, satisfaction with care, anxiety and depression, health resource utilization, and actual received care.

## Electronic supplementary material

ESM 1 (DOCX 26 kb)
