# Supplementary material for: Advance care planning in glioblastoma patients: development of a disease-specific ACP program
Source: Support Care Cancer. 2019 Jun 26;28(3):1315–24. doi: 10.1007/s00520-019-04916-9 (PMC6989589; doi:10.1007/s00520-019-04916-9)
Supplement: Supplementary file 1 — (DOCX 26 kb) [file 520_2019_4916_MOESM1_ESM.docx]

| **Supplementary File 1. Topics and questions semi-structured interview** |
| --- |

| INTRODUCTION OF THE CONCEPT   - What do you think about the concept Advance Care Planning?   PART 1: ACP IN GENERAL   - How do you feel about the tailored ACP? - Would you participate in an ACP-program? - If yes, what is the reason? If no, what is the reason? - What would you expect from such a program? - Do you think there are advantages of implementing such a program in clinical practice? - Who would benfit from such a program? In what way? And to what extent? - What are possible disadvantages of implementing such a program? - Who should lead/facilitate such a program? - How often would you like to talk about ACP topics? - What should be the maximum length for each ACP session (in minutes)? And the maximum amount of sessions? - When the ACP progam is finished, would you like to have frequent follow-up contact with the nurse practitioner? If yes, at set times or only in case you have questions?      - When in the disease trajectory would you like to discuss ACP topics? We have options: (1) shortly after diagnosis, (2) after chemoradiation, about 12-16 weeks after diagnosis, (3) after the first three courses of adjuvant chemotherapy, approximately six months after diagnosis, (4) after adjuvant chemotherapy, about nine months after diagnosis, or (5) another moment? - Could you give a rationale for this preference?     PART 2: CONTENT OF THE ACP PROGRAM  For every topic that we will propose, we would like to know if you feel this is sufficiently relevant to include in the ACP program. Please elaborate on your choice.  ***CURRENT SITUATION***   - Current health status - Expectations about the course of the disease - Roles of the patient/relatives and the physician in decision-making - Assessment of psychological distress - Prognosis - Need for spiritual support - Interaction with relatives, friends, neighbors, acquaintances and colleagues   ***WORRIES OF THE PATIENT***   - Anxieties / fears regarding the disease and treatment - Situations you don’t want to experience - Previous experiences that may cause the fear of suffering   ***POSSIBILITIES AND IMPOSSIBILITIES OF TREATMENT***   - Medical possibilities to relieve symptoms - Medical possibilities to prolong life - Futility of certain medical treatments, and reasons - Benefits and disadvantages of the medical treatments - Expectations about the impact of specific medical treatments - Burden of specific medical treatments - Consequences if a patient wants to withdraw a certain anti-tumor treatment - Consequences if a patient wants to withdraw a certain supportive treatment (for example steroids, anti-epileptic drugs, blood transfusion, antibiotics or morphine) - Consequences if a patient decides to withhold food and fluids - Resuscitation - Life support (e.g. respiration) - Presence of an advance directive - Presence of a legal proxy (i.e. someone that is appointed to make decisions on behalf of the patient in case he/she is no longer able to make decisions) - Palliative sedation - Euthanasia - Declaration on euthanasia   ***END OF LIFE PHASE***   - Preferred place of death - Conditions under which a patient can remain at home in the end-of-life phase - Measures that are necessary to stay at home in the end-of-life phase, and who can arrange this - Chance of admission to the hospital or hospice - Current arrangements for care in the end-of-life phase |
| --- |
